# The prevalence of psychological stress in student populations during the COVID-19 epidemic: a systematic review and meta-analysis

**DOI:** 10.1038/s41598-022-16328-7

**Published:** 2022-07-15

**Authors:** Yang Fang, Bo Ji, Yitian Liu, Jingyu Zhang, Qianwei Liu, Yunpeng Ge, Yana Xie, Cunzhi Liu

**Affiliations:** School of Acupuncture-Moxibustion and Tuina, Beijing University of Chinese Medicine, No. 11 North Third Ring East Road, Chaoyang District, Beijing, 100029 China

**Keywords:** Public health, Risk factors

## Abstract

Following the COVID-19 outbreak, psychological stress was particularly pronounced in the student population due to prolonged home isolation, online study, closed management, graduation, and employment pressures. The objective of this study is to identify the incidence of psychological stress reactions in student populations following a global outbreak and the associated influencing factors. Four English databases (Pubmed, Embase, Cochrane Library, Web of Science) and four Chinese biomedical databases (Chinese Biomedical Literature Database, VIP Database for Chinese Technical Periodicals, China National Knowledge Infrastructure, Wanfang) were searched in this study. We also retrieved other search engines manually. The search period was from the time of database creation to 10 March 2022. This study included cross-sectional studies related to psychological stress reactions in student populations during the COVID-19 epidemic. Three groups of researchers screened the retrieved studies and assessed the quality of the included studies using the Agency for Healthcare Research and Quality Cross-Sectional Study Quality Assessment Checklist. A random-effects model was used to analyze the prevalence of depression, anxiety, stress, and fear symptoms in the student population during the COVID-19 epidemic. Of the 146,330 records retrieved, we included 104 studies (n = 2,088,032). The quality of included studies was moderate. The prevalence of depressive symptoms in the student population during the epidemic was 32.0% (95% CI [28.0–37.0%]); anxiety symptoms was 28.0% (95% CI [24.0–32.0%]); stress symptoms was 31.0% (95% CI [23.0–39.0%]); and fear symptoms was 33.0% (95% CI [20.0–49.0%]). The prevalence differed by gender, epidemic stage, region, education stage, student major and assessment tool. The prevalence of psychological stress in the student population during the COVID-19 epidemic may be higher compared to the global prevalence of psychological stress. We need to alleviate psychological stress in the student population in a targeted manner to provide mental health services to safeguard the student population.

## Introduction

Since the outbreak of Coronavirus Disease 2019 (COVID-19), COVID-19 has rapidly spread to more than 200 countries and territories. Many countries have entered Level One Public Health Emergencies response. There were more than 500 million confirmed COVID-19 cases and more than 6 million deaths as of 17 April 2022^1^. The outbreak and expansion of the epidemic significantly affect the mental health status of the population^2^. The student population was also greatly affected by the epidemic, taking into account a variety of factors, such as prolonged home isolation, closed campus management, online learning, graduation, and employment pressures.

During serious public health emergencies, populations are more likely to experience psychological changes such as depression, anxiety, fear, and stress symptoms^3^. As a vulnerable group, students are more prone to mental health problems than people with stable incomes. The prevalence of anxiety and depressive symptoms in the Chinese student population during the Severe Acute Respiratory Syndrome (SARS) epidemic in 2003 ranged from 25.4 to 29.6%. This value was much higher than the results of the population mental health survey at that time (7.6–16.3%)^4^. Strong and persistent psychological stimuli in the student population can trigger psychological stress reactions, mainly in the form of mood changes such as depression, anxiety, stress, and fear symptoms. It can also be accompanied by symptoms such as palpitations, irritability, headaches, insomnia, and in severe cases, disruptions in the function of several systems^5^ and even lead to dependent behavior of students on alcohol, tobacco, drugs, and smartphones^6,7^. As a result, this can have a negative impact on the health and life of the student body. Therefore, mental health services and emotional stress interventions for the student population are also an important part of the fight against the COVID-19 epidemic and the promotion of future development dynamics in society.

The existing meta-analyses have either focused only on mood changes in anxiety and depression in student populations or have been limited to studies of student populations in a particular major or country^8,9^. Nevertheless, the psychological stress response in student populations is influenced by a variety of factors, such as gender, major, regional economic status, and educational stage. Moreover, the prevalence of psychological stress varies widely across studies, which greatly increases the difficulty of developing psychological intervention programs for student populations.

Our meta-analysis collected cross-sectional studies related to psychological stress in student populations globally since the onset of the epidemic to comprehensively and completely assess the psychological stress in student populations. The gender, major, academic stage, regional nuclear study phase of the epidemic, and survey approach of the student population in the study were further explored. This study was designed to provide a reference for the prevention and intervention of psychological stress reactions in student populations during the COVID-19 pandemic.

## Methods

We conducted this meta-analysis according to the PRISMA guidelines. The protocol of this study is registered in the International Prospective Register of Systematic Evaluations (*PROSPERO*), registration number CRD42020210391.

### Literature search

In this study, four Chinese databases and four English databases were searched, including the China National Knowledge Infrastructure (CNKI), Wanfang Data, CQVIP, China Biomedical Literature (SinoMed), Pubmed, Embase, Cochrane Library, and Web of Science. The search period was from the establishment of the database to March 10, 2022. According to the "PICOS" principle to formulate the search strategy, we used search terms including: “novel coronavirus pneumonia”, “NCP”, “2019-nCoV”, “COVID-19”, “coronavirus disease 2019”, “mental health”, “depression”, “anxiety”, “fear”, “stress”. The combination of subject words and free words was used in the retrieval, and the references that had been included in the literature were supplemented. In addition, we supplemented the search with relevant literature found by search engines such as Google Scholar. A detailed search strategy is provided in Supplementary Table 1.

### Inclusion and exclusion criteria

The inclusion criteria for eligible studies were: (a) the type of study included was a cross-sectional study (on-site survey or online survey); (b) the study population was the student population during the epidemic, including undergraduates, postgraduates, middle school students, and primary school students; (c) Assessing the prevalence of depression, anxiety, fear and stress symptoms using a standardized instrument or an evidence-based, self-administered scale instrument; (d) the inclusion study was conducted during the COVID-19 pandemic (since December 19, 2019). Exclusion criteria were: (a) the college or university students with mental illness already; (b) The study did not provide separate results or complete outcome data for the incidence of psychological stress in the student population.

### Data Extraction

Using a pre-designed spreadsheet, we extracted the following information from the included studies: first author, date of publication, study period, sampling method, the region where the study was conducted, sample size, characteristics of the study sample, evaluation instrument, survey method, and incidence of psychological stress (depression, anxiety, fear, stress).

### Quality assessment

We evaluated the quality of included studies using the criteria of the American Agency for Health Care Quality and Research Cross-Sectional Research Literature Quality Assessment Checklist (*AHRQ Checklist*)^10^. A total of 11 entries were available. The evaluation was done with "yes," "no," and "unclear" responses, with 0–3 being low quality, > 3–7 is medium quality, and > 7–11 being high quality.

Three groups of researchers (Yang Fang, Jingyu Zhang; Yitian Liu, Yana Xie; Yunpeng Ge, Qianwei Liu) independently performed literature screening, data extraction, and literature bias assessment. When disagreements emerged in the assessment, they were checked for discrepancies or disputes by discussing or consulting third-party solutions.

### Data synthesis and analysis

We used meta-analysis to generate pooled estimates and their 95% confidence intervals (95% CI) for the prevalence of depression, anxiety, fear, and stress symptoms in the entire sample. We used forest plots to show incidence and pooled estimates, while I^2^ tests were used to assess heterogeneity between studies. Fixed-effects models assume that the overall effect size is the same for all studies. In contrast, the random-effects model attempts to do this by assuming that the selected studies are from a larger population.^11^ When evidence heterogeneity was low (i.e., I^2^ ≤ 50 and heterogeneity *p* ≥ 0.10), a fixed-effects model was used to generate pooled estimates; otherwise, a random-effects model was used. We used subgroup analyses to explore sources of heterogeneity in the incidence of different psychological stress responses. Publication bias was assessed using funnel plots and Begg's test, as Begg's test is more applicable for large meta-analyses that include 75 or more original studies^12^. The incidence was transformed by the "PFT" method before the meta-analysis. All analyses were performed using R (version 4.2.0).

## Results

### Literature screening

**Figure 1:**
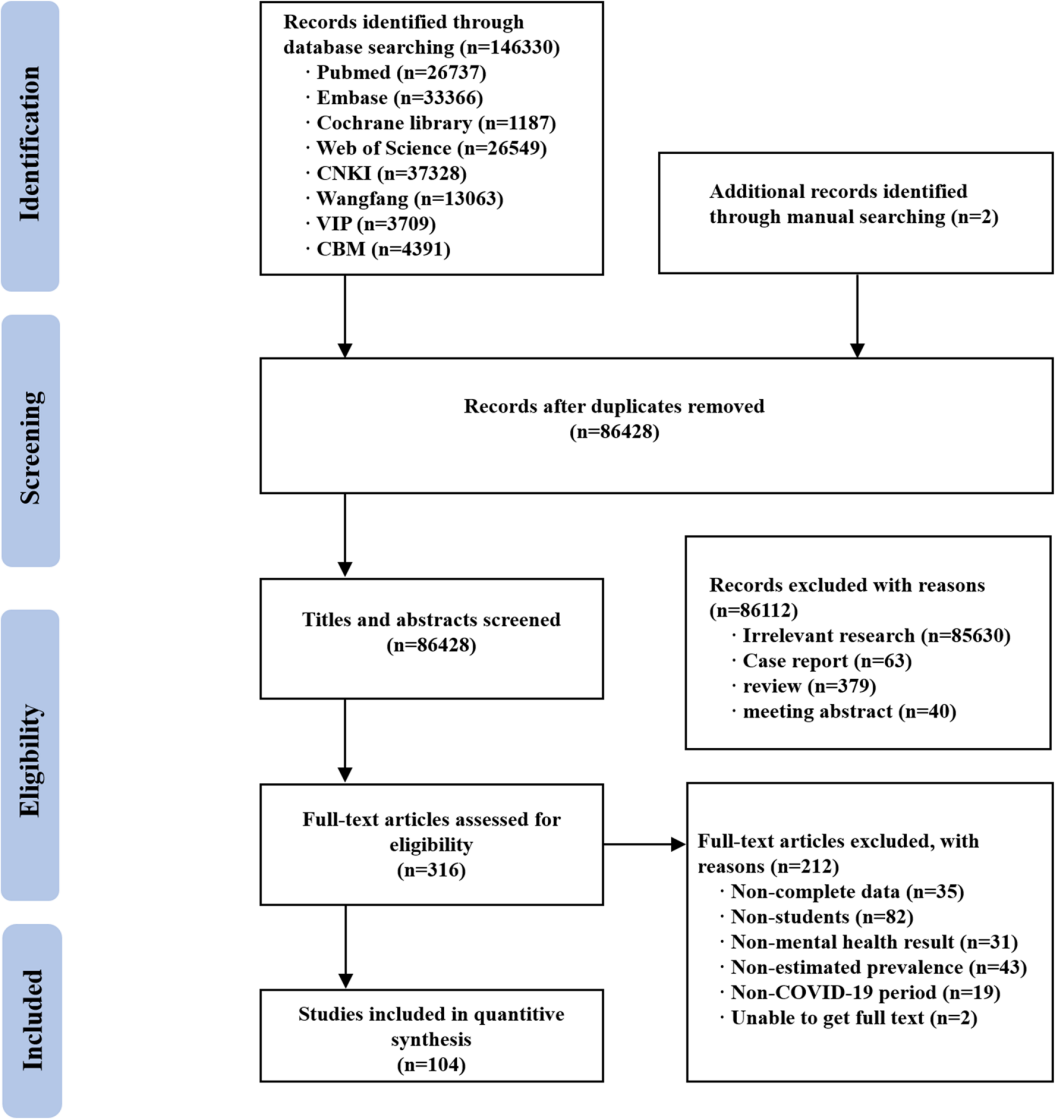
Flow diagram of the progress of acquiring the qualified literature and studies included in the meta-analysis.

Initially, 146,330 studies on this subject were searched through 8 databases and 2 studies were searched manually; subsequently, we removed 86,428 duplicate studies and 86,324 studies that did not meet the inclusion criteria for this study. A total of 104 studies were finally included in this meta-analysis^13^. The flow diagram is shown in Fig. 1.

### Study characteristics

The characteristics of the included studies are presented in Table 1. A total of 104 cross-sectional studies with 2,088,032 students were included in this study. Of these, 988,425 were males, 1,098,969 were females, and 638 were of unknown gender. Of the included studies, 75 studies reported depressive symptoms (n = 1,005,228), 93 studies reported anxiety symptoms (n = 2,048,035), 31 reported stress symptoms (n = 855,564) and 17 studies reported fear symptoms (n = 62,346). 86 studies were conducted in Asia, 8 in Europe, 5 in Africa, 1 in South America, 3 in North America, and 1 in Oceania. Regarding sampling methods, a total of 11 studies used random sampling, 3 studies used stratified sampling, 6 studies used whole group sampling, and the remaining studies used convenience sampling. Regarding the included studies, 36 studies assessed depressive symptoms using the Patient Health Questionnaire depression module-9 (PHQ-9), 8 studies assessed depressive symptoms using the Self-Rating Depression Scale (SDS); 39 studies assessed anxiety symptoms using the General Anxiety Disorder-7 Item Scale (GAD-7), 23 studies assessed anxiety symptoms using the Self-Rating Anxiety Scale (SAS); 17 studies assessed psychological stress reactions using the Depression Anxiety Stress Scale-21 Item (DASS-21), 3 studies assessed psychological stress reactions using the Symptom Checklist 90 (SCL-90), 3 studies assessed psychological stress reactions using the Hospital Anxiety and Depression Scale (HADS), and the other studies used self-administered scales or other assessment scale tools.

**Table 1:** The characteristics of 104 studies.

Study	Country	Survey time	Sampling method	Sample size (n =)	Age (year)	Gender (male/female)	Educational level	Majors	Psychological stress	Assessment tool	Investigation method
Gong Chen 2020	China	2020.5.2 ~ 2020.5.9	Handy sampling	4750	≥ 18	1652/3098	Undergraduate (4184) Postgraduate (566)	Medical	Anxiety	SAS	Questionnaire
Minjiang Ding 2020	China	2020.1	Random sampling	3055	≥ 18	1420/1635	Undergraduate (2993) Postgraduate (62)	Multiversity	Fear, Anxiety	Self-made scale	Questionnaire
Lan Gao 2020	China	2020.2.11 ~ 2020.2.16	Handy sampling	5593	21 ± 2	2290/3303	Undergraduate	Medical	Depression, Anxiety	PHQ-9, GAD-7	Questionnaire
Gaowen Yu 2020	China	NR	Random sampling	427	NR	98/329	Undergraduate	Multiversity	Depression, Anxiety	SAS, SDS	Questionnaire
Qingxiang Yu 2020	China	2020.2.9 ~ 2020.2.10	Random sampling	2074	NR	1087/987	Junior (747) Senior (1327)	/	Depression, Anxiety, fear	Self-made scale	Questionnaire
Benyu Zhang 2020	China	2020.2.6 ~ 2020.5.26	Cluster sampling	5151	≥ 18	1374/3777	Undergraduate	Multiversity	Anxiety, Fear	RQ-20, SAS	Questionnaire
Xiaolu Zhang 2020	China	2020.2	Random sampling	1486	21.69 ± 2.27	453/1033	Undergraduate (1371) Postgraduate (115)	Medical	Depression, Anxiety, Fear	PHQ-9, GAD-7, SSRS	Questionnaire
Xuehui Zhang 2020	China	2020.2.1 ~ 2020.2.8	Handy sampling	1209	21.89 ± 3.43	527/682	Undergraduate (755) Postgraduate (454)	Medical	Depression, Anxiety	PHQ-9, GAD-7	Questionnaire
Chunz Zhao 2020	China	NR	Handy sampling	376	≥ 18	73/303	Undergraduate	Multiversity	Depression, Anxiety, Fear	Self-made scale	Questionnaire
Kaiheng Zhu 2020	China	2020.2.28 ~ 2020.3.5	Random sampling	1264	NR	707/557	Primary	/	Anxiety	SCARED	Questionnaire
Xiaolin Zhu 2020	China	2020.1.30 ~ 2020.2.13	Handy sampling	1482	21 ± 3	458/1024	Senior (171) Undergraduate (1027) Postgraduate (284)	Multiversity	Depression, Anxiety, Pressure	SRQ-20, PHQ-9, GAD-7	Questionnaire
Zengli Zou 2020	China	2020.2.15 ~ 2020.2.29	Handy sampling	25,286	≥ 18	7548/17,738	Undergraduate (24,157) Postgraduate (1129)	Medical	Anxiety	SAS	Questionnaire
Erke Ke 2021	China	2020.3 ~ 2020.4	Handy sampling	7755	10.73 ± 2.98	4249/3506	Primary (5282) Junior (1728) Senior (745)	/	Anxiety	PSQ	Questionnaire
Limu Ke 2021	China	2020.2.4. ~ 2020.4.26	Handy sampling	1110	21.08 ± 1.85	395/715	Undergraduate	Medical	Depression, Anxiety	PHQ-9, GAD-7	Questionnaire
Pei Deng 2021	China	2020.2	Handy sampling	517	≥ 18	135/382	Undergraduate	Multiversity	Anxiety	SAS	Questionnaire
Jinghui Chang 2020	China	2019.1.13 ~ 2020.2.3	Handy sampling	3881	19 ~ 20	1434/2447	Undergraduate	Multiversity	Depression, Anxiety	PHQ-9, GAD-7	Questionnaire
Shushen Zheng 2020	China	NR	Handy sampling	3823	20.03 ± 1.43	1293/2530	Undergraduate	Medical	Depression, Anxiety	SAS, SDS, SSRS	Questionnaire
Wen Zhang 2021	China	2020.4 ~ 2020.5	Stratified sampling	7719	≥ 18	2686/5033	Undergraduate	Multiversity	Anxiety, Fear	Self-made scale	Questionnaire
Xi Liu 2021	China	NR	Handy sampling	1841	20.42 ± 1.70	773/1068	Undergraduate	Multiversity	Depression, Anxiety	PHQ-9, GAD-7	Questionnaire
Ya Wang 2020	China	2020.2	Handy sampling	3178	≥ 18	878/2300	Undergraduate (3170) Postgraduate (8)	Multiversity	Depression, Anxiety	HAMA, SDS	Questionnaire
Pengfei Bi 2021	China	NR	Random sampling	330	18 ~ 23	68/262	Undergraduate	Medical	Depression, Anxiety, Pressure	DASS-21	Questionnaire
Xiaopan Shi 2021	China	2020.2.25 ~ 2020.3.8	Handy sampling	1830	NR	561/1269	Undergraduate	Multiversity	Depression, Anxiety	PHQ-9, GAD-7	Questionnaire
Xingjie Yang 2020	China	2020.3.8 ~ 2020.3.15	Handy sampling	4139	≥ 18	1431/2708	Undergraduate	Multiversity	Depression, Anxiety	PHQ-9, GAD-7	Questionnaire
Dandan Shi 2022	China	2020.9	Handy sampling	7838	≥ 18	3011/4827	Undergraduate	Medical	Depression, Anxiety, Fear, Pressure	SCL-90	Questionnaire
Daokai Sun 2021	China	NR	Handy sampling	1297	≥ 18	597/700	Undergraduate	Multiversity	Anxiety	GAD-7	Questionnaire
Hongli Sun 2021	China	2020.2.6 ~ 2020.3.5	Random sampling	2597	NR	830/1767	Undergraduate	Multiversity	Fear	Self-made scale	Questionnaire
Yuelong Jin 2021	China	2020.6 ~ 2020.7	Cluster sampling	3781	20.37 ± 1.31	1950/1831	Undergraduate	Multiversity	Depression, Anxiety, Pressure	DASS-21	Questionnaire
Yan Jiang 2020	China	2020.2.27 ~ 2020.2.29	Handy sampling	339	NR	162/237	Undergraduate	Medical	Depression, Anxiety	PHQ-9, GAD-7	Questionnaire
Zhujun Jin 2021	China	2020.3	Handy sampling	569	NR	176/393	Undergraduate	Multiversity	Depression, Anxiety, Fear, Pressure	Self-made scale	Questionnaire
Yanping Li 2021	China	2020.5	Handy sampling	449	18 ~ 26	218/231	Undergraduate	Multiversity	Anxiety	SAS	Questionnaire
Hao Wang 2022	China	2020.2.23 ~ 2020.4.5	Handy sampling	3641	22.5 ± 2.35	1029/2612	Undergraduate	Multiversity	Depression, Anxiety, Pressure	DASS-21	Questionnaire
Renli Li 2020	China	2019.9 ~ 2020.4	Random sampling	2603	≥ 18	1226/1377	Undergraduate	Multiversity	Depression, Anxiety, fear	SCL-90	Questionnaire
Yue Li 2021	China	2020.2	Stratified sampling	2640	NR	824/1816	Undergraduate	Multiversity	Anxiety	SAS	Questionnaire
Peijun Liu 2021	China	2020.3.8 ~ 2020.3.14	Handy sampling	721	20.27 ± 2.87	238/483	Undergraduate (585) Postgraduate (136)	Medical	Anxiety	SAS	Questionnaire
Shuai Wang 2020	China	2020.3.8 ~ 2020.3.12	Handy sampling	1365	18 ~ 28	540/825	Undergraduate (1047) Postgraduate (318)	Multiversity	Anxiety	SAS	Questionnaire
Shaoyong Ma 2021	China	2020.2.2 ~ 2020.2.6	Handy sampling	6276	20.31 ± 1.51	1736/4540	Undergraduate	Medical	Anxiety	SAS	Questionnaire
Qianwen Qiu 2020	China	2020.2.16 ~ 2020.2.20	Handy sampling	1100	18 ~ 25	315/785	Undergraduate	Multiversity	Anxiety	SAS	Questionnaire
Jing Wang 2021	China	2020.2.18 ~ 2020.2.20	Handy sampling	840	20.16 ± 2.16	276/564	Undergraduate (795) Postgraduate (48)	Multiversity	Depression, Anxiety	SAS, SDS	Questionnaire
Nan Wu 2021	China	2020.6.9 ~ 2020.6.12	Cluster sampling	2702	20.5 ± 0.9	672/2025	Undergraduate	Medical	Depression, Anxiety	SAS, SDS	Questionnaire
Shuyin Wu 2021	China	2020.3	Handy sampling	941	21.8 ± 2.5	381/560	Undergraduate (811) Postgraduate (130)	Multiversity	Depression, Anxiety	PHQ-9, GAD-7	Questionnaire
Ruichen Jiang 2020	China	2020.2	Cluster sampling	472	NR	196/276	Undergraduate	Multiversity	Depression, Anxiety, Pressure	SCL-90	Questionnaire
Huiqi Wang 2020	China	2020.2.16 ~ 2020.2.18	Handy sampling	661	17.34 ± 1.60	305/356	Senior	/	Depression, Anxiety	PHQ-9, GAD-7	Questionnaire
Yuany Yang 2020	China	2020.2.7 ~ 2020.2.9	Handy sampling	1667	20.57 ± 2.00	803/864	Undergraduate (1546) Postgraduate (121)	Multiversity	Depression, Anxiety, fear	PQEEPH	Questionnaire
Yuanyuan Zhu 2021	China	2020.3.6 ~ 2020.4.1	Handy sampling	342	20.72 ± 1.39	45/297	Undergraduate	Medical	Depression, Anxiety	PHQ-9, GAD-7, ERQ	Questionnaire
Lina Zhao 2021	China	2020.3.20 ~ 2020.4.10	Handy sampling	666	≥ 20	262/404	Undergraduate	Medical	Depression	PHQ-9	Questionnaire
Bo Zhao 2021	China, Korea	2020.3.23 ~ 2020.4.12	Handy sampling	420	22.90 ± 3.30	133/287	Undergraduate	Multiversity	Depression	PHQ-9	Questionnaire
Yiman Huang 2021	China	2020.2 ~ 2020.3	Handy sampling	3133	20.83 ± 1.53	889/2224	Undergraduate	Multiversity	Depression, Anxiety, Pressure	DASS-21	Questionnaire
Chengqi Cao 2021	China	2020.7.13 ~ 2020.7.29	Handy sampling	57,984	14.8 ± 1.6	28,089/29,895	Junior (41,158) Senior (16,826)	/	Depression, Anxiety, Pressure	PHQ-9, GAD-7, GPS-T	Questionnaire
Xudong Zhang 2021	China	2020.2.21 ~ 2020.2.24	Handy sampling	2270	18 ~ 25	877/1393	Undergraduate	Multiversity	Depression, Anxiety, Pressure	SAS, SDS, YBOCS	Questionnaire
Yanqiu Yu 2021	China	2020.2.1 ~ 2020.2.10	Handy sampling	23,863	NR	7605/16,258	Undergraduate (23,326) Postgraduate (537)	Multiversity	Depression, Anxiety, Fear	PHQ-9	Questionnaire
Mingli Yu 2021	China	2020.3.3 ~ 2020.3.15	Handy sampling	1681	≥ 18	592/1089	Undergraduate	Multiversity	Depression	CES-D	Questionnaire
Xinli Chi 2020	China	2020.5.13 ~ 2020.5.20	Handy sampling	1794	15.26 ± 0.47	1007/787	Junior	/	Depression, Anxiety	PHQ-9, GAD-7	Questionnaire
Z.Ma 2020	China	2020.2.3 ~ 2020.2.10	Handy sampling	746,217	18 ~ 26	331,613/414,604	Undergraduate	Multiversity	Depression, Anxiety, Pressure	IES-6, PHQ-9, GAD-7	Questionnaire
Wenning Fu 2021	China	2020.5.10 ~ 2020.6.10	Handy sampling	89,588	18 ~ 30	39,194/50,394	Undergraduate	Multiversity	Anxiety	GAD-7	Questionnaire
Jincong Yu 2021	China	2020.7 ~ 2020.8	Handy sampling	9383	NR	2685/6698	Undergraduate	Multiversity	Depression	PHQ-9	Questionnaire
Juan Wang 2021	China	2020.2.4 ~ 2020.2.11	Handy sampling	538,500	6 ~ 12	287,189/251,311	Primary	/	Anxiety	GAD-7	Questionnaire
Qingqing Xu 2021	China	2020.2.4 ~ 2020.2.12	Cluster sampling	373,216	15.24 ± 1.59	193,507/179,709	Junior (244,193) Senior (129,023)	/	Anxiety	GAD-7	Questionnaire
Xiaobin Zhang 2021	China	2021.1 ~ 2021.2	Handy sampling	22,380	12 ~ 17	11,809/10,571	Junior	/	Depression, Anxiety	PHQ-9, GAD-7	Questionnaire
Yi Zhang 2021	China	2020.2.4 ~ 2020.2.12	Handy sampling	11,787	20.51 ± 1.88	5056/6731	Undergraduate	Multiversity	Depression	PHQ-9	Questionnaire
Weiwei Chang 2021	China	2019.12 ~ 2020.6	Handy sampling	4115	20.27 ± 1.30	1626/2489	Undergraduate	Medical	Depression, Anxiety, Pressure	DASS-21	Questionnaire
Mingqiang Xiang 2020	China	2020.2.25 ~ 2020.3.5	Handy sampling	1396	20.68 ± 1.84	881/515	Undergraduate (1314) Postgraduate (82)	Multiversity	Depression, Anxiety	SAS, SDS	Questionnaire
Jingyi Wang 2021	China	2020.4.16 ~ 2020.5.14	Handy sampling	6435	15.6 ± 1.7	3204/3231	Senior	/	Depression	CDI	Questionnaire
Chenyang Lin 2022	China	2020.6.12 ~ 2020.7.14	Handy sampling	1881	21.39 ± 2.48	976/905	Undergraduate (1302) Postgraduate (579)	Multiversity	Depression, Anxiety	PHQ-9, GAD-7	Questionnaire
Pei Xiao 2021	China	2020.10 ~ 2020.12	Cluster sampling	3951	19.58 ± 1.67	1674/2277	Undergraduate	Multiversity	Depression, Anxiety	PHQ-9, GAD-7	Questionnaire
Xiaolei Zheng 2021	China	2020.12.17 ~ 2020.12.19	Random sampling	954	21.1 ± 1.2	366/588	Undergraduate (877) Postgraduate (77)	Multiversity	Depression, Anxiety	PHQ-9, GAD-7	Questionnaire
Kaihan Yang 2021	China	2020.4 ~ 2020.5	Handy sampling	521	22.02 ± 1.76	117/404	Undergraduate (481) Postgraduate (40)	Multiversity	Anxiety, Fear, Pressure	SAS, SRQ-20	Questionnaire
Peng Xiong 2021	China	2020.2.20 ~ 2020.3.20	Handy sampling	563	21.52 ± 2.50	172/391	Undergraduate (456) Postgraduate(107)	Multiversity	Depression, Anxiety, Pressure	DASS-21	Questionnaire
Xiaoyan Wu 2021	China	2020.2.4 ~ 2020.2.12	Random sampling	11,787	20.45 ± 1.76	5056/6731	Undergraduate	Multiversity	Depression, Anxiety	PHQ-9, GAD-7	Questionnaire
Luke 2021	Malaysia	2020.7.1 ~ 2020.7.21	Handy sampling	316	18 ~ 31	95/221	Undergraduate	Medical	Depression, Anxiety, Pressure	DASS-21	Questionnaire
Dongfang Wang 2021	China	2020.6.1 ~ 2020.6.15	Handy sampling	8921	21.59 ± 1.81	3064/5857	Undergraduate (7428) Postgraduate (1493)	Multiversity	Depression, Anxiety, Pressure	PHQ-9, GAD-7, IES-6	Questionnaire
Villani 2021	Italy	2020.6.8 ~ 2020.7.12	Handy sampling	501	21 ~ 24	143/358	Undergraduate	Multiversity	Depression, Anxiety, Fear	SAS, SDS, PHE-2	Questionnaire
Simegn2021	Ethiopia	2020.6.30 ~ 2020.7.30	Handy sampling	423	18 ~ 34	272/151	Undergraduate	Multiversity	Depression, Anxiety, Pressure	DASS-21	Questionnaire
Xiaomei Wang 2020	America	2020.5.4 ~ 2020.5.19	Handy sampling	2031	22.88 ± 5.52	779/1252	Undergraduate (1405) Postgraduate (626)	Multiversity	Depression, Anxiety, Pressure	PHQ-9, GAD-7	Questionnaire
Sundarasen 2020	Malaysia	2020.4.20 ~ 2020.5.24	Handy sampling	983	17 ~ 25	330/653	Undergraduate (876) Postgraduate (107)	Multiversity	Anxiety	SAS	Questionnaire
Chinna 2021	Asia	2020.4 ~ 2020.5	Handy sampling	3679	NR	1519/2160	Undergraduate	Multiversity	Anxiety	SAS	Questionnaire
Karen 2021	Australia	2020.8 ~ 2020.9	Handy sampling	638	≥ 18	NR	Undergraduate	Medical	Depression, Anxiety, Pressure	DASS-21	Questionnaire
Radwan 2021	Palestine	2020.6.10 ~ 2020.7.13	Random sampling	420	10 ~ 18	137/283	Senior	/	Depression, Anxiety, Pressure	DASS-21	Questionnaire
Alsolais 2021	Saudi Arabia	2020.4.22 ~ 2020.5.16	Handy sampling	492	21.77 ± 2.47	218/274	Undergraduate	Medical	Depression, Anxiety, Pressure, Fear	DASS-21	Questionnaire
Abay 2021	Ethiopia	2020.4.15 ~ 2020.515	Handy sampling	408	≥ 18	214/194	Undergraduate	Multiversity	Depression, Anxiety, Pressure	DASS-21	Questionnaire
Ririn 2021	India	2020.4 ~ 2020.5	Stratified sampling	247	17 ~ 24	23/224	Undergraduate	Medical	Anxiety	SAS	Questionnaire
Emilijus 2021	Lithuania	2021.1.31 ~ 2021.2.7	Handy sampling	1001	20.8 ± 2.8	225/776	Undergraduate	Multiversity	Depression, Anxiety	HADS	Questionnaire
Rogowska 2021	Poland	2020.3.30 ~ 2021.6.12	Handy sampling	1961	23.23 ± 3.16	841/1120	Undergraduate (1151) Postgraduate (810)	Multiversity	Anxiety, Pressure	PSS-10, GAD-7	Questionnaire
Kristina 2021	Germany	2020.6.29 ~ 2020.7.26	Handy sampling	623	≥ 18	514/109	Undergraduate	Multiversity	Pressure	Self-made scale	Questionnaire
Kezang 2022	Bhutan	2020.9.10 ~ 2020.10.10	Handy sampling	278	21.7 ± 2.07	194/84	Undergraduate	Multiversity	Depression, Anxiety	PHQ-9, GAD-7	Questionnaire
Biswas 2021	Bengal	2020.4.21 ~ 2020.5.10	Handy sampling	425	22.0 ± 1.8	160/265	Undergraduate	Medical	Depression	PHQ-9	Questionnaire
Jesus 2021	Spain	2021.2.1 ~ 2021.3.15	Handy sampling	517	21.03 ± 4.32	409/108	Undergraduate	Multiversity	Anxiety, Fear, Pressure	FCV-19S, GAD-7, BRCS	Questionnaire
Adriana 2021	Brazil	2020.9.14 ~ 2020.10.19	Handy sampling	1224	≥ 18	384/840	Undergraduate	Multiversity	Depression, Anxiety, Pressure	DASS-21	Questionnaire
Sarah 2021	Uganda	2020.6.29 ~ 2020.7.29	Handy sampling	321	24.8 ± 5.1	198/123	Undergraduate (273) Postgraduate (48)	Multiversity	Depression, Anxiety, Pressure	DASS-21	Questionnaire
Lucia 2021	Nigeria	2020.4.29 ~ 2020.5.5	Handy sampling	386	21.0. ± 2.9	154/232	Undergraduate	Multiversity	Depression, Anxiety	HADS	Questionnaire
Chootong 2022	Thailand	2021.9 ~ 2021.10	Handy sampling	325	21 ± 3	139/186	Undergraduate	Medical	Depression, Anxiety	PHQ-9, GAD-7	Questionnaire
Mai Sakai 2022	Japan	2020.8.18 ~ 2020.10.31	Handy sampling	281	18 ~ 22	43/238	Undergraduate	Multiversity	Depression, Anxiety	HADS	Questionnaire
Puteikis 2022	Lithuania	2021.10.20 ~ 2021.11.20	Handy sampling	628	16.1 ± 1.2	186/442	senior	/	Depression, Anxiety	BDI, GAD-7,	Questionnaire
Rasma 2022	Bengal	2020.5 ~ 2020.8	Handy sampling	605	23.1 ± 3.4	245/360	Undergraduate (431) Postgraduate (174)	Multiversity	Anxiety	GAD-7	Questionnaire
Daniel 2022	Uganda	2021.6.26 ~ 2021.7.26	Handy sampling	338	≥ 18	213/125	Undergraduate (288) Postgraduate (50)	Multiversity	Anxiety	GAD-7	Questionnaire
Tiange Lu 2022	China	2020.3.19 ~ 2020.3.29	Handy sampling	795	17 ± 1.42	582/213	Senior	/	Depression, Anxiety	SAS, SDS	Questionnaire
Maria 2022	Mexico	NR	Handy sampling	252	21.12 ± 3.21	86/166	Undergraduate	Multiversity	Depression, Anxiety, Pressure	DASS-21	Questionnaire
Mohammad 2022	Bengal	2021.1.7 ~ 2021.3.27	Handy sampling	731	≥ 18	355/376	Undergraduate	Medical	Depression, Anxiety, Pressure	DASS-21	Questionnaire
Scott 2021	America	2020.4.13 ~ 2020.4.28	Handy sampling	1428	22.3 ± 9.0	476/952	Undergraduate (1400) Postgraduate (28)	Medical	Depression, Anxiety	PHQ-9, GAD-7	Questionnaire
Kyoko 2021	Japan	2020.5.20 ~ 2020.6.16	Handy sampling	2449	20.5 ± 3.5	1330/1119	Undergraduate	Multiversity	Depression	PHQ-9	Questionnaire
Hakami 2021	Saudi Arabia	2020.4.14 ~ 2020.4.26	Handy sampling	697	21.76 ± 1.86	316/381	Undergraduate	Medical	Depression, Anxiety, Pressure	DASS-21	Questionnaire
Thomas 2021	Switzerland	2020.3 ~ 2020.9	Handy sampling	3571	26.0 ± 5.5	1089/2482	Undergraduate	Multiversity	Depression	PHQ-9	Questionnaire
Abdullah 2021	Saudi Arabia	2020.4.21 ~ 20,205.20	Random sampling	119	NR	101/18	Undergraduate	Multiversity	Anxiety	GAD-7	Questionnaire
Benojir 2021	Bengal	2020.4.23 ~ 2020.4.30	Handy sampling	1317	≥ 18	766/551	Undergraduate (846) Postgraduate (471)	Multiversity	Depression, Anxiety, Fear	GAD-7, FCS-19S, WHO-5	Questionnaire
Beata 2022	Czech	2020.1 ~ 2020.6	Handy sampling	3099	≥ 18	955/2144	Undergraduate	Multiversity	Depression, Anxiety	PHQ-15, GAD-7	Questionnaire

*SAS* Self-rating anxiety scale, *PHQ-9* Patient health questionnaire depression module-9, *GAD-7* General anxiety disorder-7 item scale, *SDS* Self-rating depression scale, *RQ-20* Relationship questionnaire-20, *SSRS* Social Support rating scale, *SCARED* The screen for child anxiety related emotional disorders, *SRQ-20* Self-reporting questionnaire-20, *HAMA* Hamilton anxiety scale, *DASS-21* Depression anxiety stress scale-21 item, *SCL-90* Symptom checklist 90, *PQEEPH* Psychological questionnaires for emergent events of public health, ERQ Emotion regulation questionnaire, *GPS-T* Global pain scale-T, *YBOCS* Yale-brown obsessive–compulsive scale, *CES-D* Center for epidemiological survey-depression scale, *IES-6* Impact of event scale-revised, *CDI* Children’s depression inventory, *PHE-S* Psychometric hepatic encephalopathy score, HADS Hospital anxiety and depression scale, *FCV-19S* Fear of COVID-19 scale, *BDI* Beck depression rating scale, / Not reported.

### Study quality

Among the included studies, a total of 8 studies had a quality score of “0–3”, 78 studies had a quality score of “4–7”, and 18 studies had a quality score of “8–11”. The quality of the included studies was moderate. The specific evaluations are shown in Table 2.

**Table 2:** Quality rating of included studies using the criteria of the American Agency for Health Care Quality and Research Cross-Sectional Research Literature Quality Assessment Checklist (AHRQ Checklist).

Study	Define the information score	List inclusion and exclusion criteria for exposed and unexposed participants (cases and controls) or refer to previous publications	Indicate time period used for identifying patients	Indicate whether participants were consecutive if not population	Indicate if evaluators of subjective components of study were masked to other aspects of the status of the participants	Describe any assessments undertaken for quality assurance purposes	Explain any patient exclusions from analysis	Describe how confounding variables were assessed and/or controlled	If applicable, explain how missing data were handled in the analysis	Summarise patients’ response rates and completeness of data collection	Clarify what follow-up, if any, was expected and the percentage of patients with incomplete data	Total score
Gong Chen 2020	Yes	No	Yes	Yes	No	No	Yes	Yes	No	Yes	Unclear	6
Minjiang Ding 2020	Yes	No	Yes	Yes	Yes	Yes	Yes	No	Yes	Yes	Unclear	8
Lan Gao 2020	Yes	No	Yes	Yes	No	Yes	Yes	No	No	Yes	Unclear	6
Gaowen Yu 2020	Yes	No	No	Yes	No	No	Yes	No	No	No	Unclear	3
Qingxiang Yu 2020	Yes	Yes	Yes	Yes	No	No	Yes	No	No	No	Unclear	5
Benyu Zhang 2020	Yes	Yes	Yes	Yes	No	Yes	Yes	Yes	No	Yes	Unclear	8
Xiaolu Zhang 2020	Yes	Yes	Yes	Yes	No	No	Yes	No	No	Yes	Unclear	7
Xuehui Zhang 2020	Yes	No	Yes	Yes	Yes	Yes	Yes	Yes	No	Yes	Unclear	8
Chunz Zhao 2020	Yes	No	No	Yes	No	No	No	No	No	Yes	Unclear	3
Kaiheng Zhu 2020	Yes	Yes	Yes	Yes	No	No	No	No	No	Yes	Unclear	5
Xiaolin Zhu 2020	Yes	Yes	Yes	Yes	No	No	Yes	Yes	No	Yes	Unclear	7
Zengli Zou 2020	Yes	Yes	Yes	Yes	No	Yes	Yes	Yes	No	Yes	Unclear	8
Erke Ke 2021	Yes	Yes	Yes	Yes	No	No	Yes	Yes	No	Yes	Unclear	7
Limu Ke 2021	Yes	Yes	Yes	Yes	Yes	Yes	No	No	No	Yes	Unclear	7
Pei Deng 2021	Yes	No	Yes	Yes	No	No	Yes	No	No	Yes	Unclear	5
Jinghui Chang 2020	Yes	No	Yes	Yes	No	No	No	No	No	Yes	Unclear	4
Shushen Zheng 2020	Yes	No	No	Yes	No	No	No	No	No	Yes	Unclear	3
Wen Zhang 2021	Yes	Yes	Yes	Yes	Yes	Yes	No	Yes	No	Yes	Unclear	8
Xi Liu 2021	Yes	No	No	Yes	No	No	Yes	Yes	No	Yes	Unclear	5
Ya Wang 2020	Yes	No	Yes	Yes	No	No	Yes	No	No	Yes	Unclear	5
Pengfei Bi 2021	Yes	No	No	Yes	No	No	Yes	No	No	Yes	Unclear	4
Xiaopan Shi 2021	Yes	Yes	Yes	Yes	No	No	No	No	No	Yes	Unclear	5
Xingjie Yang 2020	Yes	Yes	Yes	Yes	No	No	Yes	No	No	Yes	Unclear	6
Dandan Shi 2022	Yes	Yes	Yes	Yes	No	No	Yes	No	No	Yes	Unclear	6
Daokai Sun 2021	Yes	Yes	No	Yes	No	Yes	Yes	No	Yes	Yes	Unclear	7
Hongli Sun 2021	Yes	Yes	Yes	Yes	No	Yes	Yes	No	No	Yes	Unclear	7
Yuelong Jin 2021	Yes	Yes	Yes	Yes	No	No	Yes	No	No	Yes	Unclear	6
Yan Jiang 2020	Yes	Yes	Yes	Yes	No	No	Yes	No	No	Yes	Unclear	6
Zhujun Jin 2021	Yes	No	Yes	Yes	No	No	Yes	No	No	Yes	Unclear	5
Yanping Li 2021	Yes	No	Yes	No	No	No	No	No	No	Yes	Unclear	3
Hao Wang 2022	Yes	Yes	Yes	Yes	No	Yes	Yes	No	Yes	Yes	Unclear	8
Renli Li 2020	Yes	No	Yes	Yes	No	No	Yes	No	No	Yes	Unclear	5
Yue Li 2021	Yes	Yes	Yes	Yes	No	No	Yes	No	No	Yes	Unclear	6
Peijun Liu 2021	Yes	Yes	Yes	Yes	No	Yes	Yes	No	No	Yes	Unclear	7
Shuai Wang 2020	Yes	No	Yes	Yes	No	No	Yes	No	No	Yes	Unclear	5
Shaoyong Ma 2021	Yes	Yes	Yes	Yes	No	Yes	Yes	Yes	No	Yes	Unclear	8
Qianwen Qiu 2020	Yes	No	Yes	Yes	No	Yes	Yes	Yes	No	Yes	Unclear	7
Jing Wang 2021	Yes	Yes	Yes	Yes	No	No	Yes	No	No	Yes	Unclear	6
Nan Wu 2021	Yes	No	Yes	Yes	No	No	Yes	No	No	Yes	Unclear	5
Shuyin Wu 2021	Yes	Yes	Yes	Yes	No	No	Yes	No	No	Yes	Unclear	6
Ruichen Jiang 2020	Yes	Yes	Yes	Yes	No	Yes	Yes	Yes	No	Yes	Unclear	8
Huiqi Wang 2020	Yes	No	Yes	Yes	No	No	Yes	No	No	Yes	Unclear	5
Yuany Yang 2020	Yes	Yes	Yes	Yes	No	Yes	Yes	Yes	No	Yes	Unclear	8
Yuanyuan Zhu 2021	Yes	Yes	Yes	Yes	No	Yes	Yes	No	No	Yes	No	7
Lina Zhao 2021	Yes	Yes	Yes	Yes	No	No	Yes	No	No	Yes	Unclear	6
Bo Zhao 2021	Yes	Yes	Yes	Yes	No	Yes	Yes	Yes	No	Yes	No	8
Yiman Huang 2021	Yes	No	Yes	Yes	No	No	Yes	No	No	Yes	No	5
Chenqi Cao 2021	Yes	No	Yes	Yes	No	No	Yes	No	No	Yes	Unclear	5
Xudong Zhang 2021	Yes	Yes	Yes	Yes	No	No	Yes	No	No	Yes	No	6
Yanqiu Yu 2021	Yes	Yes	Yes	Yes	No	Yes	Yes	No	No	Yes	Unclear	7
Mingli Yu 2021	Yes	No	Yes	Yes	No	No	Yes	No	No	Yes	Unclear	5
Xinli Chi 2020	Yes	Yes	Yes	Yes	No	Yes	Yes	No	Yes	Yes	Unclear	8
Z.Ma 2020	Yes	No	Yes	Yes	No	Yes	Yes	No	No	Yes	Unclear	6
Wenning Fu 2021	Yes	No	Yes	Yes	No	No	Yes	No	No	Yes	Unclear	5
Jincong Yu 2021	Yes	Yes	Yes	Yes	No	Yes	Yes	No	Yes	Yes	No	8
Juan Wang 2021	Yes	Yes	Yes	Yes	No	No	Yes	No	No	Yes	Unclear	6
Qingqing Xu 2021	Yes	Yes	Yes	Yes	No	Yes	Yes	No	No	Yes	No	7
Xiaobin Zhang 2021	Yes	Yes	Yes	Yes	No	No	Yes	No	No	Yes	No	6
Yi Zhang 2021	Yes	Yes	Yes	Yes	No	No	Yes	No	No	Yes	No	6
Weiwei Chang 2021	Yes	Yes	Yes	Yes	No	Yes	Yes	No	Yes	Yes	No	8
Mingqiang Xiang 2020	Yes	Yes	No	Yes	No	No	Yes	No	No	Yes	No	5
Jingyi Wang 2021	Yes	Yes	Yes	Yes	No	Yes	Yes	No	No	Yes	Unclear	7
Chenyang Lin 2022	Yes	Yes	No	Yes	No	No	Yes	No	No	Yes	Unclear	5
Pei Xiao 2021	Yes	Yes	Yes	Yes	No	Yes	Yes	No	No	Yes	Unclear	7
Xiaolei Zheng 2021	Yes	Yes	Yes	Yes	No	No	Yes	No	No	Yes	Unclear	6
Kaihan Yang 2021	Yes	Yes	Yes	Yes	No	No	Yes	No	No	Yes	Unclear	6
Peng Xiong 2021	Yes	Yes	Yes	Yes	No	Yes	Yes	No	Yes	Yes	Unclear	8
Xiaoyan Wu 2021	Yes	Yes	Yes	Yes	No	No	Yes	No	No	Yes	Unclear	6
Luke 2021	Yes	Yes	Yes	Yes	No	No	Yes	No	Yes	Yes	Unclear	7
Dongfang Wang 2021	Yes	Yes	No	Yes	No	No	Yes	No	No	Yes	Unclear	5
Villani 2021	Yes	Yes	Yes	Yes	No	No	Yes	No	No	Yes	Unclear	6
Simegn 2021	Yes	Yes	Yes	Yes	No	Yes	Yes	No	Yes	Yes	Unclear	8
Xiaomei Wang 2020	Yes	Yes	No	Yes	No	No	Yes	No	No	Yes	Unclear	5
Sundarasen 2020	Yes	Yes	Yes	Yes	No	No	Yes	No	No	Yes	Unclear	6
Chinna 2021	Yes	Yes	Yes	Yes	No	Yes	Yes	No	Yes	Yes	No	8
Karen 2021	Yes	No	No	Yes	No	No	Yes	No	No	No	No	3
Radwan 2021	Yes	Yes	Yes	Yes	No	No	Yes	No	Yes	Yes	No	7
Alsolais 2021	Yes	Yes	Yes	Yes	No	No	Yes	No	No	Yes	No	6
Abay 2021	Yes	Yes	No	Yes	No	No	Yes	No	No	Yes	No	5
Ririn 2021	Yes	Yes	Yes	Yes	No	No	Yes	No	No	Yes	Unclear	6
Emilijus 2021	Yes	Yes	Yes	Yes	No	No	Yes	No	No	Yes	Unclear	6
Rogowska 2021	Yes	Yes	Yes	Yes	No	Yes	Yes	No	Yes	Yes	Unclear	8
Kristina 2021	Yes	Yes	Yes	Yes	No	Yes	Yes	No	Yes	Yes	Unclear	8
Kezang 2022	Yes	Yes	No	Yes	No	Yes	No	No	No	Yes	Unclear	5
Biswas 2021	Yes	Yes	Yes	Yes	No	Yes	No	No	No	Yes	Unclear	6
Jesus 2021	Yes	Yes	Yes	Yes	No	Yes	Yes	No	No	Yes	Unclear	7
Adriana 2021	Yes	Yes	Yes	Yes	No	No	Yes	No	No	Yes	Unclear	6
Sarah 2021	Yes	Yes	Yes	Yes	No	No	Yes	No	Yes	Yes	Unclear	7
Lucia 2021	Yes	Yes	Yes	Yes	No	No	Yes	No	No	Yes	Unclear	6
Chootong 2022	Yes	No	No	Yes	No	No	Yes	No	No	Yes	No	4
Mai Sakai 2022	Yes	Yes	No	Yes	No	No	Yes	No	No	Yes	No	5
Puteikis 2022	Yes	Yes	No	Yes	No	No	Yes	No	No	Yes	No	5
Rasma 2022	Yes	Yes	Yes	Yes	No	Yes	Yes	No	No	Yes	Unclear	7
Daniel 2022	Yes	Yes	No	Yes	No	No	Yes	No	No	Yes	Unclear	5
Tiange Lu 2022	Yes	Yes	Yes	Yes	No	No	Yes	No	No	Yes	Unclear	6
Maria 2022	Yes	No	No	Yes	No	No	No	No	No	Yes	Unclear	3
Mohammad 2022	Yes	Yes	Yes	Yes	No	No	Yes	No	No	Yes	Unclear	6
Scott 2021	Yes	Yes	Yes	Yes	No	No	Yes	No	No	Yes	Unclear	6
Kyoko 2021	Yes	Yes	No	Yes	No	No	Yes	No	No	Yes	Unclear	5
Hakami 2021	Yes	Yes	Yes	Yes	No	No	Yes	No	Yes	Yes	Unclear	7
Thomas 2021	Yes	No	No	Yes	No	No	No	No	No	Yes	Unclear	3
Abdullah 2021	Yes	No	No	Yes	No	No	No	No	No	Yes	Unclear	3
Benojir 2021	Yes	Yes	Yes	Yes	No	No	Yes	No	Yes	Yes	No	7
Beata 2022	Yes	Yes	Yes	Yes	No	No	Yes	No	No	Yes	Unclear	6

### The pooled prevalence of depressive symptom

**Figure 2:**
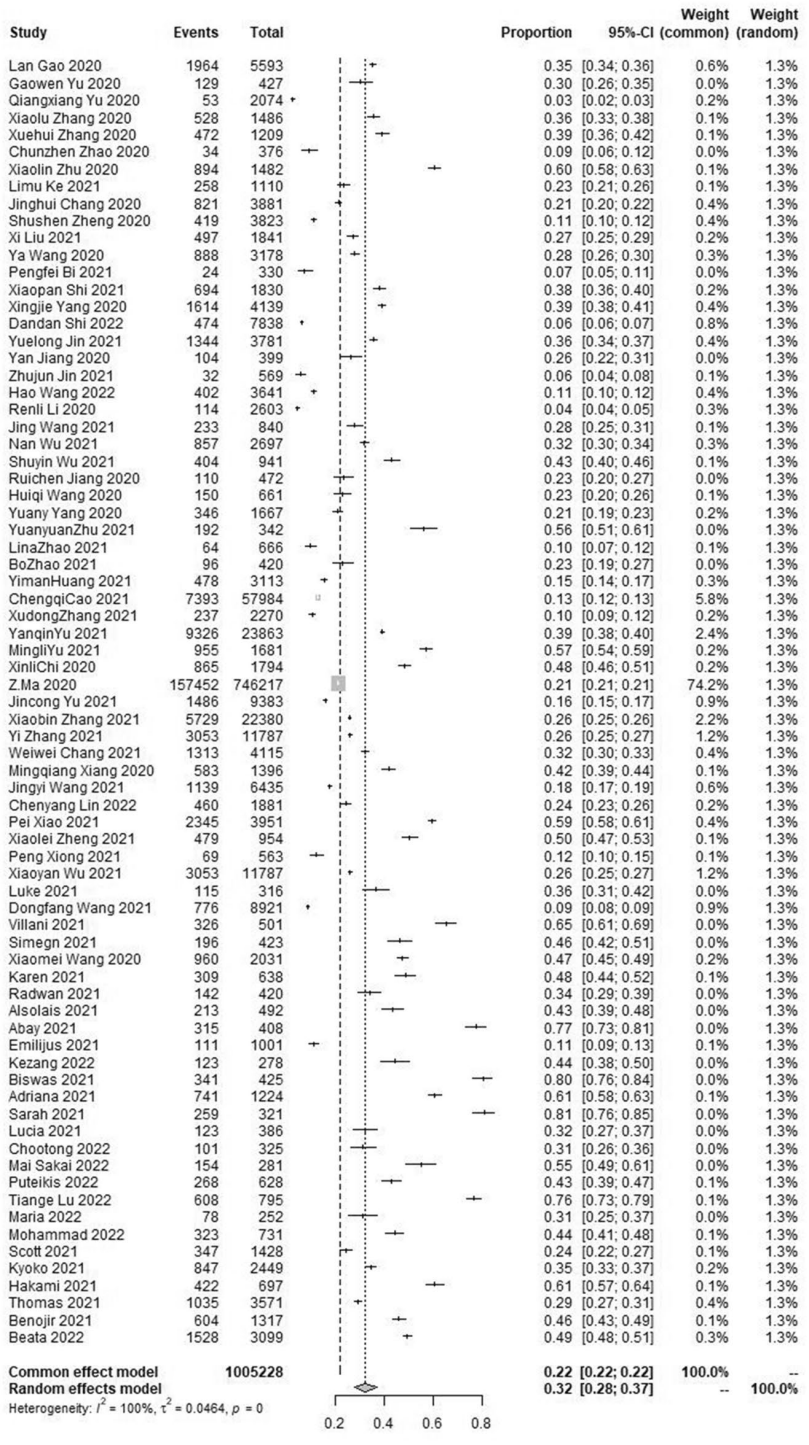
Forest plot of the meta-analysis on prevalence rates of depressive symptoms in the student population.

The results of the meta-analysis showed that the pooled prevalence of depressive symptoms in the student population was 32.0% with high heterogeneity (95% CI [28.0 ~ 37.0%], I^2^ = 100%, *p* < 0.001; Fig. 2). No statistically significant publication bias was found in the included 75 studies by Begg’s test (*p* = 0.6116 > 0.05). Sensitivity analysis results showed no obvious change in effect values when single studies were excluded one by one and then subjected to Meta-analysis, suggesting more stable study results.

### The pooled prevalence of anxiety symptom

**Figure 3:**
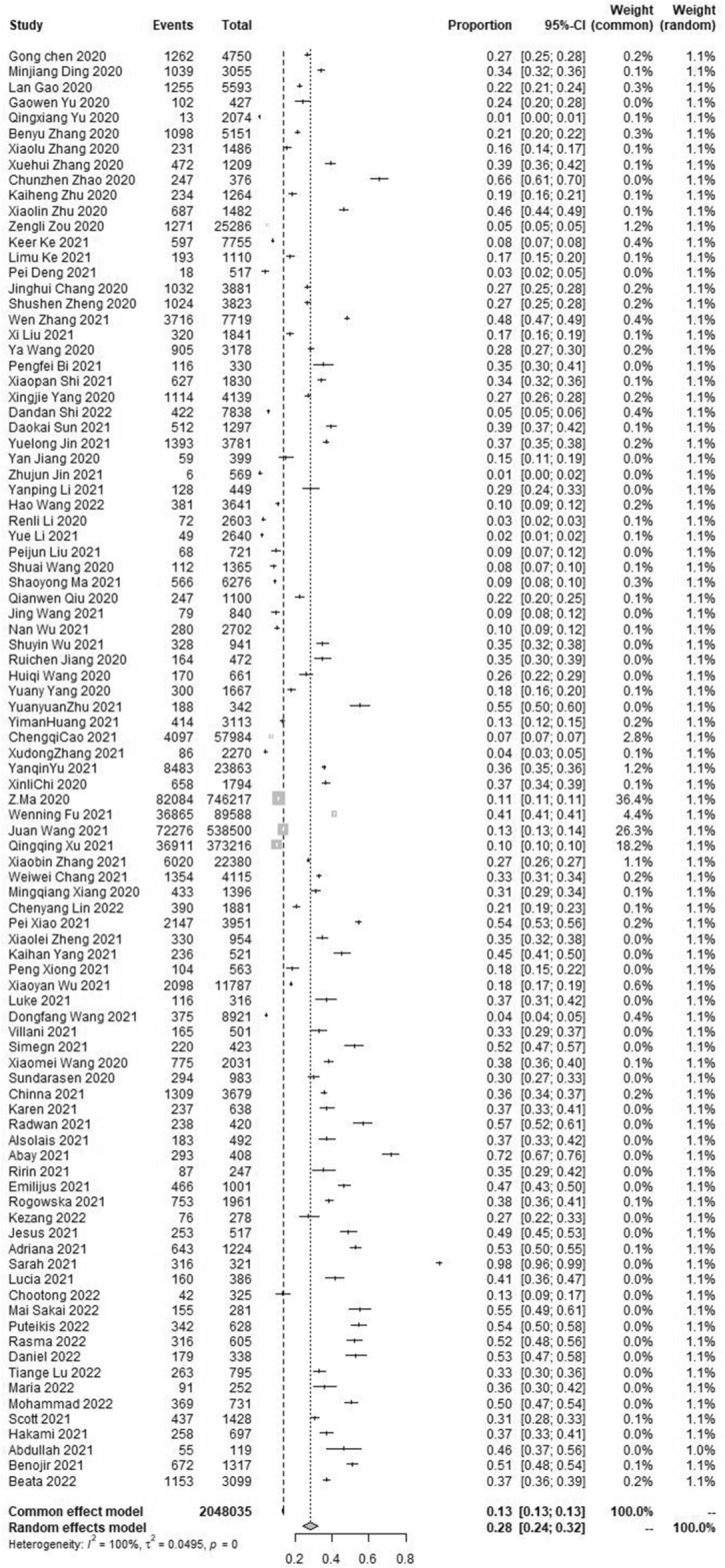
Forest plot of the meta-analysis on prevalence rates of anxiety symptoms in the student population.

The results of the meta-analysis showed that the pooled prevalence of anxiety symptoms in the student population was 28.0% with high heterogeneity (95% CI [24.0 ~ 32.0%], I^2^ = 100%, *p* < 0.001; Fig. 3). No statistically significant publication bias was found in the included 93 studies by Begg’s test (*p* = 0.9233 > 0.05). Sensitivity analysis results showed no obvious change in effect values when single studies were excluded one by one and then subjected to Meta-analysis, suggesting more stable study results.

### The pooled prevalence of stress symptom

**Figure 4:**
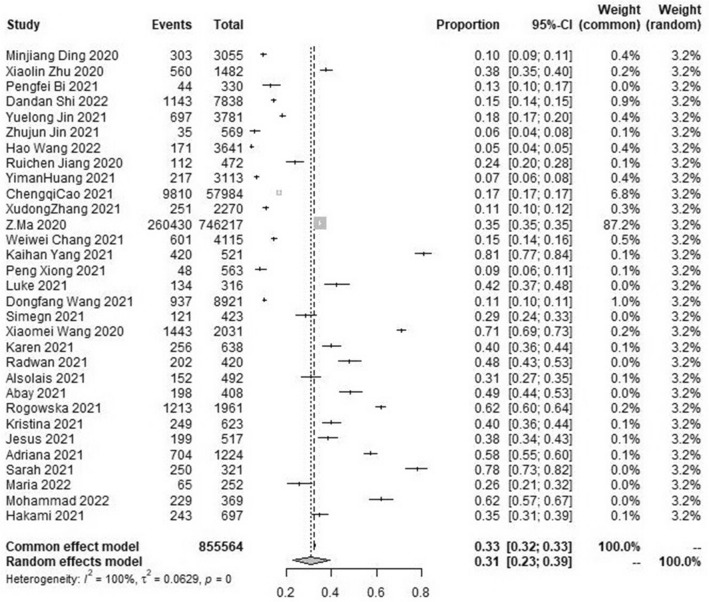
Forest plot of the meta-analysis on prevalence rates of pressure symptoms in the student population.

The results of the meta-analysis showed that the pooled prevalence of stress symptom in the student population was 31.0% with high heterogeneity (95% CI [23.0 ~ 39.0%], I^2^ = 100%, *p* < 0.001; Fig. 4). No statistically significant publication bias was found in the included 31 studies by Begg’s test (*p* = 0.1430 > 0.05). Sensitivity analysis results showed no obvious change in effect values when single studies were excluded one by one and then subjected to Meta-analysis, suggesting more stable study results.

### The pooled prevalence of fear symptom

**Figure 5:**
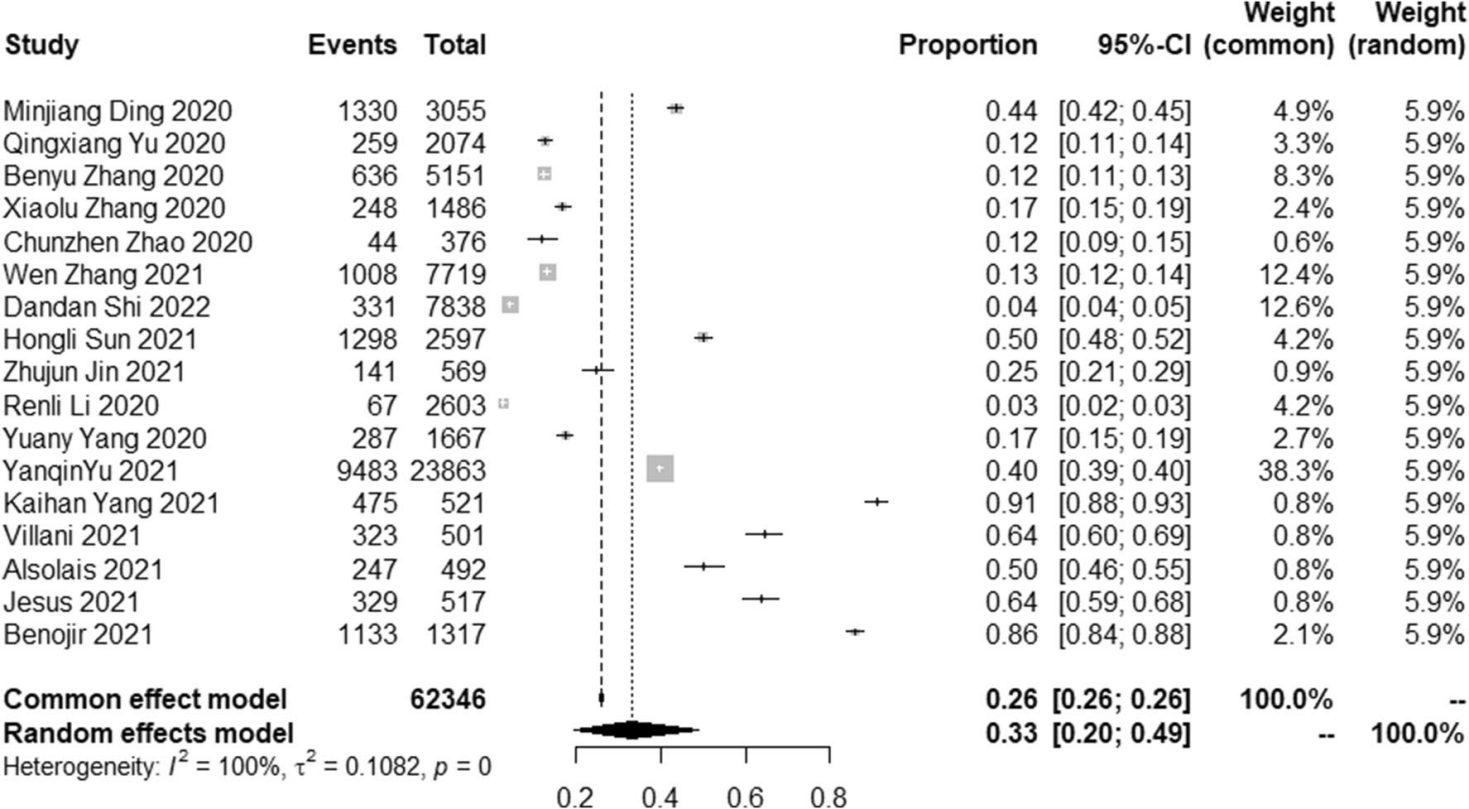
Forest plot of the meta-analysis on prevalence rates of fear symptoms in the student population.

The results of the meta-analysis showed that the pooled prevalence of fear symptoms in the student population was 33.0% with high heterogeneity (95% CI [20.0 ~ 49.0%], I^2^ = 100%, *p* < 0.001; Fig. 5). The Begg’s test found statistically significant publication bias in the 17 included studies (*p* = 0.0238 < 0.05). Sensitivity analysis results showed no obvious change in effect values when single studies were excluded one by one and then subjected to Meta-analysis, suggesting more stable study results.

### Subgroup analysis

Subgroup analysis showed that the pooled prevalence of depression, anxiety, stress, and fear symptoms in the student population was influenced by gender, the period of the epidemic, the region, the stage of education, the student’s major, and the instrument used in the evaluation.

The prevalence of depression (36.0%, 95% CI [28.0–44.0%]), anxiety (27.0%, 95% CI [21.0–33.0%]), and stress (19.0%, 95% CI [12.0–28.0%]) symptoms was higher among females than males in the student population. Among the geographic regions, the prevalence of psychological stress in the student population was lower in Eastern Asia than in other regions. For students at different educational levels, the prevalence of depressive symptoms and anxiety symptoms were higher in undergraduate and postgraduate students than in primary school and middle school students, while the prevalence of stress symptoms was the same in undergraduate and postgraduate students as in middle school students. In addition, non-medical students had higher prevalence of depression, anxiety, and stress symptoms than medical students. It is noteworthy that as the epidemic progressed from the early outbreak phase to the current "normalized" management phase, the incidence of psychological stress in the student population increased rather than decreased. All details of the subgroup analysis are shown in Table 3.

**Table 3:** Subgroup analysis of psychological stress responses in the student population during COVID-19.

a. Subgroup analysis of the incidence of depression						
Variable	k	Proportion	95% CI	I^2^	τ^2^	*p*
**Gender**						
Male	30	0.32	[0.26 ~ 0.39]	100%	0.0394	*p* = 0
Female	30	0.36	[0.28 ~ 0.44]	100%	0.0564	*p* = 0
**Research period**						
Early stage of COVID-19 outbreak (2019.12 ~ 2020.5)	45	0.31	[0.26 ~ 0.37]	100%	0.0403	*p* = 0
The normalization stage of COVID-19 (2020.6 ~ Now)	27	0.35	[0.28 ~ 0.43]	100%	0.0452	*p* = 0
**Sample source region**						
Eastern Asia	52	0.27	[0.23 ~ 0.32]	100%	0.0325	*p* = 0
Western Asia	4	0.46	[0.35 ~ 0.57]	96%	0.0120	*p* < 0.01
Southern Asia	5	0.48	[0.30 ~ 0.65]	98%	0.0406	*p* < 0.01
Europe	5	0.38	[0.20 ~ 0.58]	99.7%	0.0507	*p* < 0.01
North America	3	0.34	[0.21 ~ 0.48]	99%	0.0155	*p* < 0.01
South America	1	0.61	[0.58 ~ 0.63]	NA	NA	NA
Africa	4	0.60	[0.36 ~ 0.82]	99%	0.0618	*p* < 0.01
Oceania	1	0.48	[0.44 ~ 0.52]	NA	NA	NA
**Educational stage**						
Undergraduate and Postgraduate	65	0.33	[0.28 ~ 0.38]	100%	0.0429	*p* = 0
Middle school	9	0.28	[0.20 ~ 0.35]	100%	0.0169	*p* = 0
**Major**						
Medical	29	0.33	[0.26 ~ 0.40]	100%	0.0391	*p* = 0
Non-medical	30	0.39	[0.33 ~ 0.45]	100%	0.0299	*p* = 0
**Evaluation tool**						
PHQ-9	36	0.33	[0.28 ~ 0.38]	100%	0.0279	*p* = 0
SDS	8	0.35	[0.20 ~ 0.53]	100%	0.0673	*p* = 0
DASS-21	16	0.37	[0.26 ~ 0.49]	100%	0.0578	*p* = 0
SCL-90	2	0.13	[0.01 ~ 0.34]	99%	0.0325	*p* < 0.01
HADS	3	0.31	[0.09 ~ 0.58]	99%	0.0640	*p* < 0.01
Self-made scale	4	0.25	[0.18 ~ 0.33]	95%	0.0080	*p* < 0.01

b. Subgroup analysis of the incidence of anxiety						
Variable	k	Proportion	95% CI	I^2^	τ^2^	*p*
**Gender**						
Male	37	0.24	[0.19 ~ 0.29]	100%	0.0332	*p* = 0
Female	37	0.27	[0.21 ~ 0.33]	100%	0.0423	*p* = 0
**Research period**						
Early stage of COVID-19 outbreak (2019.12 ~ 2020.5)	61	0.24	[0.20 ~ 0.29]	100%	0.0401	*p* = 0
The normalization stage of COVID-19 (2020.6 ~ Now)	25	0.37	[0.27 ~ 0.47]	100%	0.0674	*p* = 0
**Sample source region**						
Eastern Asia	64	0.22	[0.18 ~ 0.26]	100%	0.0388	*p* = 0
Western Asia	4	0.44	[0.35 ~ 0.54]	94%	0.0085	*p* < 0.01
Southern Asia	8	0.36	[0.27 ~ 0.47]	98%	0.0222	*p* < 0.01
Europe	6	0.43	[0.37 ~ 0.49]	95%	0.0064	*p* < 0.01
North America	3	0.35	[0.30 ~ 0.40]	91%	0.0017	*p* < 0.01
South America	1	0.53	[0.50 ~ 0.55]	NA	NA	NA
Africa	5	0.67	[0.41 ~ 0.88]	99%	0.0855	*p* < 0.01
Oceania	1	0.37	[0.33 ~ 0.41]	NA	NA	NA
**Educational stage**						
Undergraduate and Postgraduate	80	0.29	[0.25 ~ 0.33]	100%	0.0487	*p* = 0
Middle school	9	0.25	[0.12 ~ 0.40]	100%	0.0658	*p* = 0
Primary school	2	0.15	[0.10 ~ 0.22]	97%	0.0036	*p* < 0.01
**Major**						
Medical	34	0.25	[0.20 ~ 0.30]	100	0.0307	*p* = 0
Non-medical	31	0.41	[0.33 ~ 0.49]	100	0.0491	*p* = 0
**Evaluation tool**						
GAD-7	39	0.30	[0.25 ~ 0.35]	100%	0.0291	*p* = 0
SAS	23	0.19	[0.14 ~ 0.25]	100%	0.0302	*p* = 0
DASS-21	17	0.42	[0.31 ~ 0.54]	100%	0.0639	*p* = 0
SCL-90	3	0.11	[0.00 ~ 0.34]	99%	0.0621	*p* < 0.01
HADS	3	0.48	[0.40 ~ 0.55]	84%	0.0038	*p* < 0.01
Self-made scale	5	0.23	[0.03 ~ 0.56]	100%	0.1531	*p* = 0

c. Subgroup analysis of the incidence of pressure						
Variable	k	Proportion	95% CI	I^2^	τ^2^	*p*
**Gender**						
Male	11	0.16	[0.12 ~ 0.21]	96%	0.0090	*p* < 0.01
Female	11	0.19	[0.12 ~ 0.28]	99%	0.0268	*p* < 0.01
**Research period**						
Early stage of COVID-19 outbreak (2019.12 ~ 2020.5)	15	0.29	[0.17 ~ 0.43]	100%	0.0827	*p* = 0
The normalization stage of COVID-19 (2020.6 ~ Now)	14	0.35	[0.25 ~ 0.47]	100%	0.0495	*p* = 0
**Sample source region**						
Eastern Asia	15	0.18	[0.10 ~ 0.28]	100%	0.0504	*p* = 0
Western Asia	3	0.38	[0.28 ~ 0.48]	93%	0.0079	*p* < 0.01
Southern Asia	2	0.52	[0.33 ~ 0.71]	96%	0.0188	*p* < 0.01
Europe	3	0.47	[0.32 ~ 0.62]	99%	0.0173	*p* < 0.01
North America	2	0.48	[0.09 ~ 0.89]	99%	0.1093	*p* < 0.01
South America	1	0.58	[0.55 ~ 0.60]	NA	NA	NA
Africa	3	0.52	[0.24 ~ 0.79]	99%	0.0665	*p* < 0.01
Oceania	1	0.40	[0.36 ~ 0.44]	NA	NA	NA
**Educational stage**						
Undergraduate and Postgraduate	28	0.31	[0.22 ~ 0.40]	100%	0.0676	*p* = 0
Middle school	2	0.31	[0.06 ~ 0.64]	99%	0.0583	*p* < 0.01
**Major**						
Medical	9	0.28	[0.18 ~ 0.40]	99%	0.0349	*p* < 0.01
Non-medical	15	0.41	[0.29 ~ 0.54]	100%	0.0661	*p* = 0
**Evaluation tool**						
SRQ-20	2	0.60	[0.18 ~ 0.94]	100%	0.1018	*p* < 0.01
IES-6	2	0.21	[0.03 ~ 0.49]	100%	0.0456	*p* = 0
DASS-21	17	0.31	[0.21 ~ 0.42]	100%	0.0584	*p* = 0
SCL-90	2	0.19	[0.11 ~ 0.29]	96%	0.0033	*p* < 0.01
Self-made scale	3	0.17	[0.02 ~ 0.40]	99%	0.0536	*p* < 0.01

d. Subgroup analysis of the incidence of fear						
Variable	k	Proportion	95% CI	I^2^	τ^2^	*p*
**Research period**						
Early stage of COVID-19 outbreak (2019.12 ~ 2020.5)	13	0.34	[0.18 ~ 0.51]	100%	0.1084	*p* = 0
The normalization stage of COVID-19 (2020.6 ~ Now)	3	0.40	[0.05 ~ 0.84]	100%	0.1730	*p* = 0
**Sample source region**						
Eastern Asia	13	0.24	[0.12 ~ 0.39]	100%	0.0864	*p* = 0
Western Asia	2	0.70	[0.31 ~ 0.96]	100%	0.0796	*p* < 0.01
Europe	2	0.64	[0.61 ~ 0.67]	0%	0.0000	*p* = 0.78

## Discussion

Since the outbreak of the epidemic, COVID-19 has spread rapidly to many countries and regions. As a vulnerable group in the population, the COVID-19 epidemic not only threatens the life and health of the student population but also triggers multiple psychological stress reactions. By identifying the types of students' psychological stress reactions and understanding the influence of relevant factors on the incidence of students' psychological stress reactions, this study can better help us identify individuals in the student population who are more likely to experience psychological stress reactions and develop relevant mental health intervention plans in a targeted manner.

### Occurrence of psychological stress in student populations

Our study found that the pooled prevalence of depression, anxiety, stress, and fear symptoms in the student population during the COVID-19 outbreak was 32.0, 28.0%, 31.0, and 33.0%. Related studies reported that the prevalence of depression, anxiety, and stress symptoms in the general population during the New Coronation epidemic were 28.0, 26.9, and 8.1%^14,15^. This result suggests that the prevalence of psychological stress in the student population during the New Coronation epidemic was slightly higher than that in the general population. We also found differences in the incidence of psychological stress reactions due to factors such as students' country of residence, stage of education, stage of the epidemic, profession, and the instruments evaluated in the studies. For instance, some studies collected samples only from student populations in medical schools^16^; others conducted sampling only in primary and secondary schools^17^; and others sampled only in a fixed area of a particular country^18^, etc. These differences in study design may be the main source of heterogeneity. Overall, the student population had a higher incidence of psychological stress during the COVID-19 outbreak than before the outbreak^19,20^.

### Vulnerable populations of psychological stress among students

From the subgroup analysis of several predictors identified in the study, we found a greater effect of gender, educational stage, and student major on the incidence of psychological stress reactions in students.

#### Female student population

Our study revealed that the prevalence of psychological stress in the female student population during the COVID-19 epidemic was much higher in depression (36.0%), anxiety (27.0%), and stress (19.0%) symptoms than in males students. This suggests that the female student population is more prone to psychological. Even before the COVID-19 outbreak, the prevalence of symptoms such as depression and anxiety was significantly higher in female than in the male population^21,22^. Females are more emotionally expressive than males, their mental and emotional states are more susceptible to external factors than males, and females show different neurobiological responses when exposed to stressors^23,24^. Psychological and physiological differences between females and males may provide a basis for the finding that female student populations are more prone to psychological stress reactions.

#### Undergraduate and postgraduate student population

Our study found that the undergraduate and postgraduate student population also exhibited a higher prevalence of psychological stress during the epidemic, which is consistent with previous research findings^25^. The reasons for this outcome are multi-layered: on the one hand, a large proportion of undergraduate and postgraduate students may not be able to return to school because of the epidemic. Reduced learning efficiency in distance online education, prolonged lack of social activities, postponement of relevant professional exams, delayed academic progress and pressure to graduate may have caused them to suffer additional psychological and emotional distress^26^; On the other hand, most the undergraduate and postgraduate students are resident on campus, and the long-term effects of the epidemic have left them with much less opportunity to see their families; In addition, the unemployment and unpredictability caused by the COVID-19 pandemic will cause additional strain on graduating undergraduate and postgraduate students.

#### Non-medical student population

Previous studies have reported higher prevalence of psychological stress among medical students compared to the social population during the COVID-19 epidemic^8,27^. Our study found that non-medical students exhibited higher levels of depression, anxiety, and stress symptoms compared to medical majors. We speculate that this may be because medical students are more knowledgeable about COVID-19 and are relatively less susceptible to news and internet information about COVID-19^28,29^; medical students can apply what they have learned to self-regulate and reduce the level of psychological stress; medical students can also use what they have learned to participate in the prevention and control of the COVID-19 outbreak by helping to alleviate the psychological stress of their surrounding housemates, classmates or colleagues^30^. In addition, most medical students' families are relatively well-off and will be less affected by the epidemic, which makes medical students worry-free in this regard. This result suggests that we should pay more attention to mental health issues of non-medical students and provide education and counseling with knowledge about COVID-19.

#### African and South American Student population

Our study found that psychological stress occurs more severely in student populations in Africa and South America than in other regions. Regional social conditions such as poor economic status, low education, and unemployment are important risk factors for triggering psychological stress during the COVID-19 pandemic^31^. The relatively tight medical resources, the high socioeconomic impact of the epidemic shock, and the dissemination of information related to COVID-19 contributed to the significantly higher incidence of psychological stress among students in these regions.

### Rehabilitation of students’ psychological stress in the “post-epidemic era”

Our study revealed a different result from previous research. Psychological stress in the student population increased rather than decreased during the "normalization" phase of the epidemic compared to the early outbreak phase^9,32^. This result suggests that the factors influencing the psychological stress response of the student population may be multidimensional and multifaceted, not only limited to the severity of the epidemic but also influenced by the students' family situation, graduation and employment pressures, personal exposure to concentrated isolation and uncertainty of information related to the epidemic^33^. Although the epidemic is not as severe at this stage as it was during the initial outbreak, mental health problems persist in the student population. We should pay more attention to the recovery of the mental health of the student population in the "post-epidemic era" and develop targeted mental health assessments and intervention programs for students. These evaluations and interventions include Internet cognitive behavioral therapy, personal psychoneuroimmune prevention, and Chinese music therapy, among others^34,35^.

## Strengths and limitations

This study systematically and comprehensively collected studies related to psychological stress reactions in student populations worldwide since the onset of the pandemic, to provide a more complete assessment of psychological stress reactions in student populations since the onset of COVID-19, and to analyze the relevant influencing factors and susceptible populations of psychological stress reactions in student populations. This can provide a reference for the development of prevention and intervention programs to address psychological stress in student populations during a global pandemic.

The following problems remain in this study: first, the included studies were mainly focused on the Asian region, with a small number of studies from other regions, which makes the assessment of the incidence of psychological stress in student populations across global regions somewhat biased and limits the generalizability of the findings; second, although we assessed the possible sources of heterogeneity through subgroup analysis, the incidence of psychological stress in student populations still there was a high level of heterogeneity, and this heterogeneity may be due to unidentified relevant factors that need to be further studied and explored; third, the majority of the included studies had a moderate quality rating. Based on the quality evaluation of the literature we suggest that more attention should be paid to the quality control of studies in future studies, especially for the treatment of confounding influences, the treatment of missing data, and the reporting of follow-up; fourth, although we conducted appropriate analyses of psychological stress in the student population during the epidemic, there were differences in the participants in the study and future longitudinal data are needed to examine the psychological stress response symptoms in the student population during the epidemic.; fifth, this meta-analysis could not determine the effect of COVID-19 infection on the psychological stress response of the student population because we did not include separate cohorts of students infected with COVID-19 and those not infected with COVID-19 in each study; finally, few of the included studies described or compared mental health services or related interventions, which prevented us from exploring which interventions better alleviated psychological stress symptoms in the student population.

Both now and in the future, when the epidemic is still prevalent, it is critical to identify the psychological stress profile of the student population and the associated influencing factors and to develop targeted mental health interventions. Future research should focus on interventions and protection against the onset of psychological stress in student populations, identify effective treatments, and develop targeted mental health service plans.

## Conclusion

Our study showed a significant increase in the prevalence of depression, anxiety, stress, and fear symptoms in the student population during the COVID-19 epidemic. Psychological stress was more pronounced in female students, undergraduate students, graduate students, and non-medical students. This suggests that a series of effective measures should be taken by individuals, families, schools, society, and government to target and alleviate the psychological stress reactions of the student population and to provide mental health service protection for the student population.

## Supplementary Information

Supplementary Information 1.

Supplementary Information 2.

## Data Availability

The datasets provided in this study can be found in online databases. The names and URLs of the databases are in the supplementary material of the article.
