# Supplementary material for: The prevalence of psychological stress in student populations during the COVID-19 epidemic: a systematic review and meta-analysis
Source: Sci Rep. 2022 Jul 15;12:12118. doi: 10.1038/s41598-022-16328-7 (PMC9284967; doi:10.1038/s41598-022-16328-7)
Supplement: Supplementary file 1 — Supplementary Information 1. [file 41598_2022_16328_MOESM1_ESM.docx]

**Supplementary Figure 1. Funnel diagram**

**a. Incidence of depressive
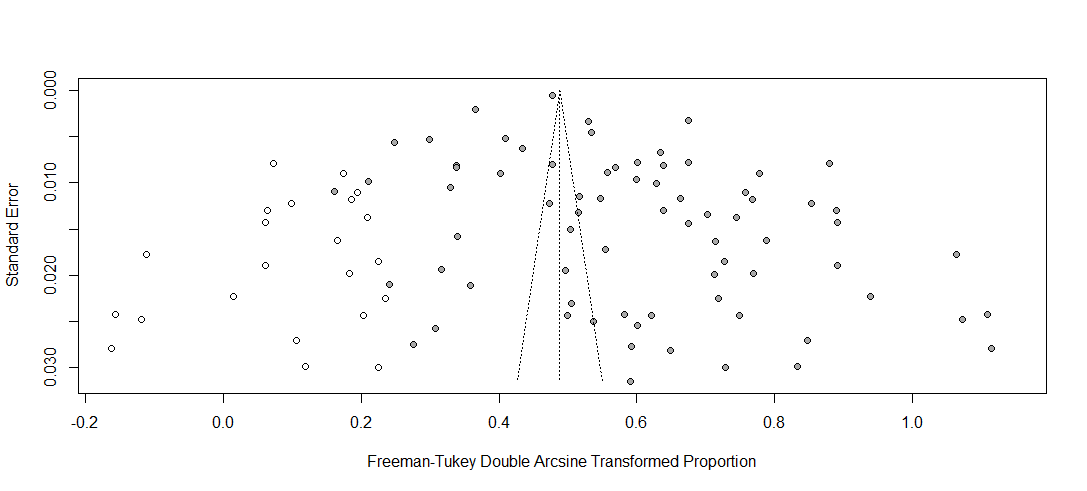
**

**b. Incidence of anxiety**

**
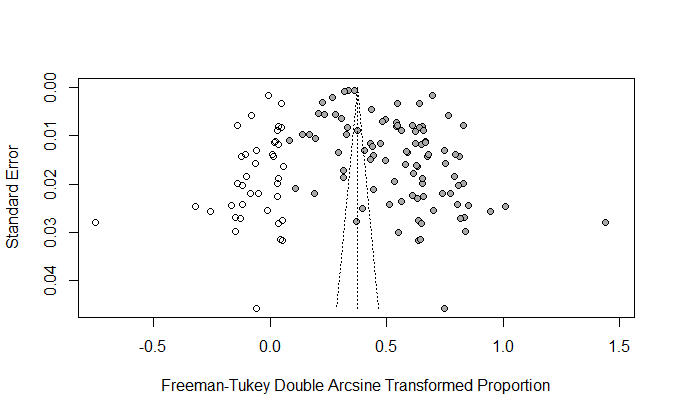
**

**c. Incidence of pressure**

**
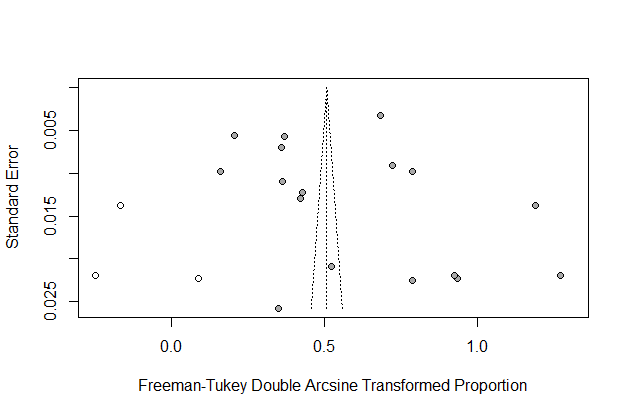
**

**d. Incidence of fear
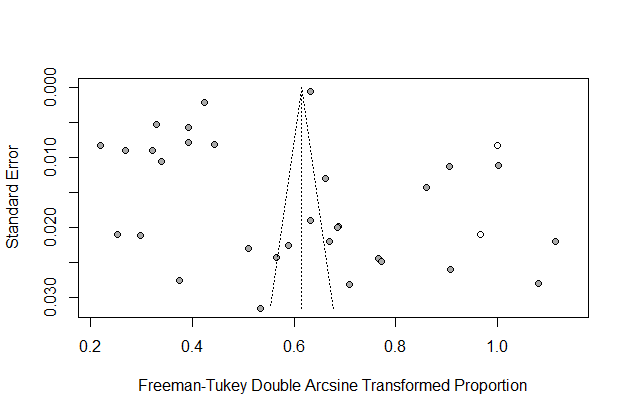
**
