# Supplementary material for: The prevalence of psychological stress in student populations during the COVID-19 epidemic: a systematic review and meta-analysis
Source: Sci Rep. 2022 Jul 15;12:12118. doi: 10.1038/s41598-022-16328-7 (PMC9284967; doi:10.1038/s41598-022-16328-7)
Supplement: Supplementary file 2 — Supplementary Information 2. [file 41598_2022_16328_MOESM2_ESM.docx]

**Supplementary Table 1. Literature search strategies**

| Databases | Search strategies | Publication date |
| --- | --- | --- |
| Chinese language |  |  |
| China National Knowledge Infrastructure | ( ( ( ( ( (主题=新型冠状病毒肺炎 OR 题名=新型冠状病毒肺炎 OR v_subject=中英文扩展(新型冠状病毒肺炎) OR title=中英文扩展(新型冠状病毒肺炎)) OR (主题=新冠病毒 OR 题名=新冠病毒 OR v_subject=中英文扩展(新冠病毒) OR title=中英文扩展(新冠病毒))) OR ( (主题=新冠肺炎 OR 题名=新冠肺炎 OR v_subject=中英文扩展(新冠肺炎) OR title=中英文扩展(新冠肺炎)) OR (主题=中英文扩展(COVID-19) OR 题名=中英文扩展(COVID-19) OR v_subject=COVID-19 OR title=COVID-19) ) ) OR ( (主题=2019冠状病毒病 OR 题名=2019冠状病毒病 OR v_subject=中英文扩展(2019冠状病毒病) OR title=中英文扩展(2019冠状病毒病)) OR (主题=2019新型冠状病毒感染 OR 题名=2019新型冠状病毒感染 OR v_subject=中英文扩展(2019新型冠状病毒感染) OR title=中英文扩展(2019新型冠状病毒感染)) ) ) OR ( (主题=2019-nCOV肺炎 OR 题名=2019-nCOV肺炎 OR v_subject=中英文扩展(2019-nCOV肺炎) OR title=中英文扩展(2019-nCOV肺炎)) OR (主题=新型冠状病毒 OR 题名=新型冠状病毒 OR v_subject=中英文扩展(新型冠状病毒) OR title=中英文扩展(新型冠状病毒)) ) ) OR (主题=中英文扩展(NCP) 题名=中英文扩展(NCP) OR v_subject=NCP OR title=NCP) ) AND ( ( ( ( ( (主题=抑郁 OR 题名=抑郁 OR v_subject=中英文扩展(抑郁) OR title=中英文扩展(抑郁)) OR (主题=焦虑 OR 题名=焦虑 OR v_subject=中英文扩展(焦虑) OR title=中英文扩展(焦虑)) ) OR ( (主题=恐惧 OR 题名=恐惧 OR v_subject=中英文扩展(恐惧) OR title=中英文扩展(恐惧)) OR (主题=压力 或者 题名=压力 OR v_subject=中英文扩展(压力) 或者 title=中英文扩展(压力)) ) ) OR ( (主题=心理 OR 题名=心理 OR v_subject=中英文扩展(心理) OR title=中英文扩展(心理)) OR (主题=应激 OR 题名=应激 OR v_subject=中英文扩展(应激) OR title=中英文扩展(应激)) ) ) OR ( (主题=危机 OR 题名=危机 OR v_subject=中英文扩展(危机) OR title=中英文扩展(危机)) OR (主题=失眠 OR 题名=失眠 OR v_subject=中英文扩展(失眠) OR title=中英文扩展(失眠)) ) ) OR ( (主题=创伤 OR 题名=创伤 OR v_subject=中英文扩展(创伤) OR title=中英文扩展(创伤)) OR (主题=失眠障碍 OR 题名=失眠障碍 OR v_subject=中英文扩展(失眠障碍) OR title=中英文扩展(失眠障碍)) ) ) | inception to to March 10, 2022 |
| Wanfang data | ( 主题:("新型冠状病毒肺炎") or 主题:("新冠病毒") or 主题:("新冠肺炎") or 主题:("COVID-19") or 主题:("2019冠状病毒病") or 主题:("2019新型冠状病毒感染") or 主题:("2019-nCOV肺炎") or 主题:("新型冠状病毒") or 主题:("NCP")) AND (主题:("抑郁") or 主题:("焦虑") or 主题:("恐惧") or 主题:("压力") or 主题:("心理") or 主题:("应激") or 主题:("危机") or 主题:("失眠") or 主题:("睡眠障碍") or 主题:("创伤")) | inception to to March 10, 2022 |
| CQVIP | ((M=新型冠状病毒肺炎) OR (M=新冠病毒) OR (M=新冠肺炎) OR (M=COVID-19) OR (M=2019冠状病毒病) OR (M=2019新型冠状病毒感染) OR (M=2019-nCOV肺炎) OR (M=新型冠状病毒) OR (M= NCP)) AND ((M=抑郁) OR (M=焦虑) OR (M=恐惧) OR (M=压力) OR (M=心理) OR (M=应激) OR (M=危机) OR (M=失眠) OR (M=睡眠障碍) OR (M=创伤)) | inception to to March 10, 2022 |
| SionMed | ("焦虑"[常用字段:智能] OR "抑郁"[常用字段:智能] OR "恐惧"[常用字段:智能] OR "压力"[常用字段:智能] OR "心理应激"[常用字段:智能] OR "危机"[常用字段:智能] OR "失眠"[常用字段:智能] OR "睡眠障碍"[常用字段:智能] OR "创伤"[常用字段:智能]) AND ("新型冠状病毒肺炎"[常用字段:智能] OR "新冠病毒"[常用字段:智能] OR "新冠肺炎"[常用字段:智能] OR "COVID-19"[常用字段:智能] OR "2019冠状病毒病"[常用字段:智能] OR "2019新型冠状病毒感染"[常用字段:智能] OR "2019-nCOV肺炎"[常用字段:智能] OR "新型冠状病毒"[常用字段:智能] OR "NCP"[常用字段:智能]) | inception to to March 10, 2022 |
| English language |  |  |
| PubMed | #1 "COVID-19"[Mesh] OR "SARS-CoV-2"[Mesh]  #2 (((("Depression"[Mesh]) OR "Depressive Disorder"[Mesh]) OR "Anxiety"[Mesh]) OR "Stress Disorders, Traumatic, Acute"[Mesh]) OR "Fear"[Mesh]  #3 ((((novel coronavirus pneumonia[Title/Abstract]) OR (NCP[Title/Abstract])) OR (2019-nCoV[Title/Abstract])) OR (COVID-19[Title/Abstract])) OR (coronavirus disease 2019[Title/Abstract])  #4 (((((mentalhealth[Title/Abstract]) OR (depression[Title/Abstract])) OR (anxiety[Title/Abstract])) OR (psycho*[Title/Abstract])) OR (stress[Title/Abstract])) OR (fear[Title/Abstract])  #5 #1 OR #3  #6 #2 OR #4  #7 #5 AND #6 | inception to to March 10, 2022 |
| Embase | #1 'coronavirus disease 2019'/exp OR 'severe acute respiratory syndrome coronavirus 2'/exp  #2 'depression'/exp OR 'anxiety'/exp OR 'physiological stress'/exp OR 'fear'/exp  #3 'novel coronavirus pneumonia':ab,ti OR ncp:ab,ti OR '2019 ncov':ab,ti OR 'covid 19':ab,ti OR 'coronavirus disease 2019':ab,ti  #4 mentalhealth:ab,ti OR depression:ab,ti OR anxiety:ab,ti OR psycho*:ab,ti OR stress:ab,ti OR fear:ab,ti  #5 #1 OR #3  #6 #2 OR #4  #7 #5 AND #6 | inception to to March 10, 2022 |
| Cochrane Library | #1 MeSH descriptor: [COVID-19] explode all trees  #2 MeSH descriptor: [SARS-CoV-2] explode all trees  #3 MeSH descriptor: [Depression] explode all trees  #4 MeSH descriptor: [Anxiety] explode all trees  #5 MeSH descriptor: [Fear] explode all trees  #6 (novel coronavirus pneumonia):ti.ab.kw OR (NCP):ti.ab.kw OR (2019 nCoV):ti.ab.kw OR (COVID-19):ti.ab.kw OR (coronavirus disease 2019):ti.ab.kw  #7 (mentalhealth):ti.ab.kw OR (depression):ti.ab.kw OR (anxiety):ti.ab.kw OR (stress):ti.ab.kw OR (fear):ti.ab.kw  #8 #1 OR #2 OR #6  #9 #3 OR #4 OR #5 OR #7  #10 #8 AND #9 | inception to to March 10, 2022 |
| Web of Science | (TS=mentalhealth OR TS=depression OR TS=anxiety OR TS=psycho* OR TS=stress OR TS=fear) AND (TS=novel coronavirus pneumonia OR TS=NCP OR TS=2019-nCoV OR TS=COVID-19 OR TS=coronavirus disease 2019 OR TS=SARS-CoV-2) | inception to to March 10, 2022 |
